# Supplementary material for: ACE2-Independent Interaction of SARS-CoV-2 Spike Protein with Human Epithelial Cells Is Inhibited by Unfractionated Heparin
Source: Cells. 2021 Jun 7;10(6):1419. doi: 10.3390/cells10061419 (PMC8229176; doi:10.3390/cells10061419)
Supplement: Supplementary file 1 [file cells-10-01419-s001.zip › cells-1242119-supplementary.pdf]

Supporting information

Supplementary Table S1. Primer Sequences

Primer	Sequence (5’-3’)
ACE2 Forward	GGGATCAGAGATCGGAAGAAGAAA
ACE2 Reverse	AGGAGGTCTGAACATCATCAGTG
TMPRSS2 Forward	TAGTGTCCCCAGCCTACCTC
TMPRSS2 Reverse	GCACCAAGGGCACTGTCTAT
GAPDH Forward	TGCACCACCAACTGCTTAGC
GAPDH Reverse	GGCATGGACTGTGGTCATGAG

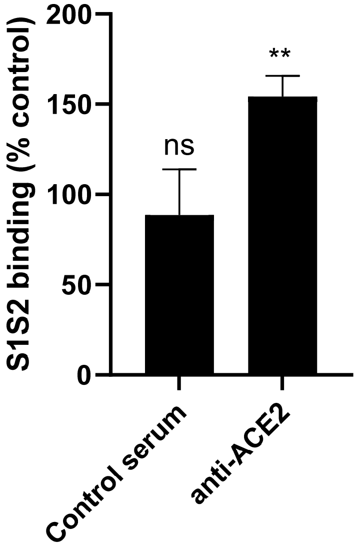

Supplementary Figure S1. wtS1S2 binding to RT4 cells is not inhibited by anti-hACE2 antibody. RT4 cells were incubated with 10ug/ml anti-human ACE2 monoclonal antibody for 30 min at 37°C, then with 33nM wtS1S2 for a further 60 min at 37°C. After a further 60 min at 37°C, cells were washed and fluorescent secondary anti-His6 antibody added for a further 30 min at 21°C. Cell-associated fluorescence was measured by flow cytometry and are shown as a percentage of the wtS1S2 attachment to untreated control cells. Data are means +/- SEM from 4 separate experiments performed in duplicate. The significance of the difference from 100 was assessed by a one sample t test; ns = not significant; ** $p<0.001$.

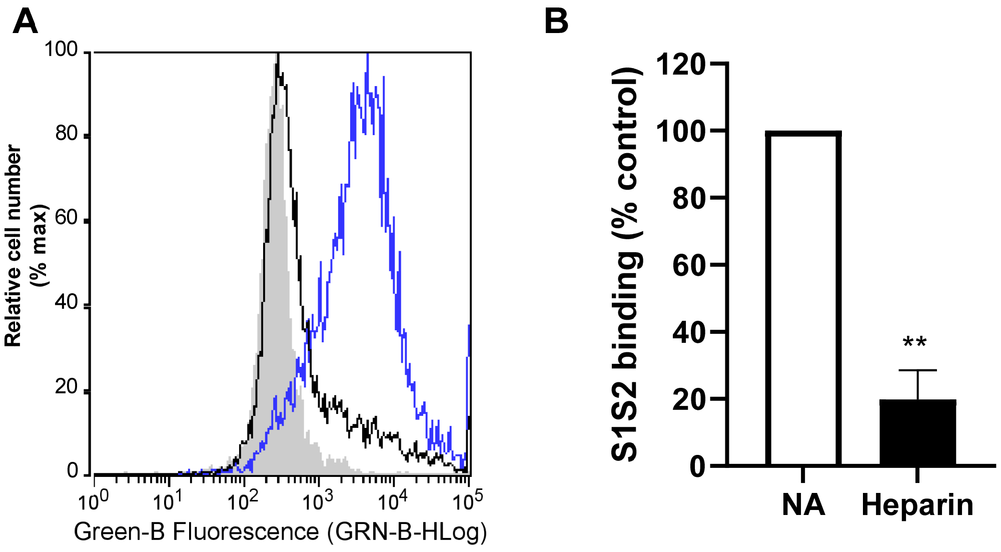

Supplementary Figure S2. Unfractionated heparin inhibits wtS1S2 binding to RT4 cells. RT4 cells were incubated with 10U/ml unfractionated heparin for 30 min at 37°C, then with 33nM wtS1S2 for a further 60 min at 37°C. After a further 60 min at 37°C, cells were washed and fluorescent secondary anti-His6 antibody added for a further 30 min at 21°C. Cell-associated fluorescence was measured by flow cytometry. A) representative histogram showing wtS1S2 binding to untreated cells (blue), heparin-treated cells (black) or secondary only control (solid grey). B) Mean +/- SEM from 3 separate experiments performed in duplicate, shown as a percentage of the wtS1S2 attachment to untreated control cells (NA). The significance of the difference from 100 was assessed by a one sample t test; ** $p<0.001$.
